# Correction: Skeleton interoception regulates bone and fat metabolism through hypothalamic neuroendocrine NPY

**DOI:** 10.7554/eLife.85738

**Published:** 2023-01-03

**Authors:** Xiao Lv, Feng Gao, Tuo Peter Li, Peng Xue, Xiao Wang, Mei Wan, Bo Hu, Hao Chen, Amit Jain, Zengwu Shao, Xu Cao

**Keywords:** Mouse

Lv X, Gao F, Li TP, Xue P, Wang X, Wan M, Hu B, Chen H, Jain A, Shao Z, Cao X. 2021. Skeleton interoception regulates bone and fat metabolism through hypothalamic neuroendocrine NPY. *eLife* **10**:e70324. doi: 10.7554/eLife.70324.

Published 1 September 2021

We are correcting an error where we mistakenly included two repeat images in Panel 4 G (second row), 5 J (second row) and 7 A (first and third in top row). The repeated images come from same batch of experiments that show co-staining with the same antibodies (perilipin & osteocalcin; YFP & osterix). The slides were possibly mixed during the assembly of the figures, which may have been in part due to the similarities of the images between these groups. The authors have now corrected the error by using the correct image for each group. There is not any other change in the main text of the paper.

The corrections are shown below:

The corrected Figure5 (Panel J):

**Figure 1:**
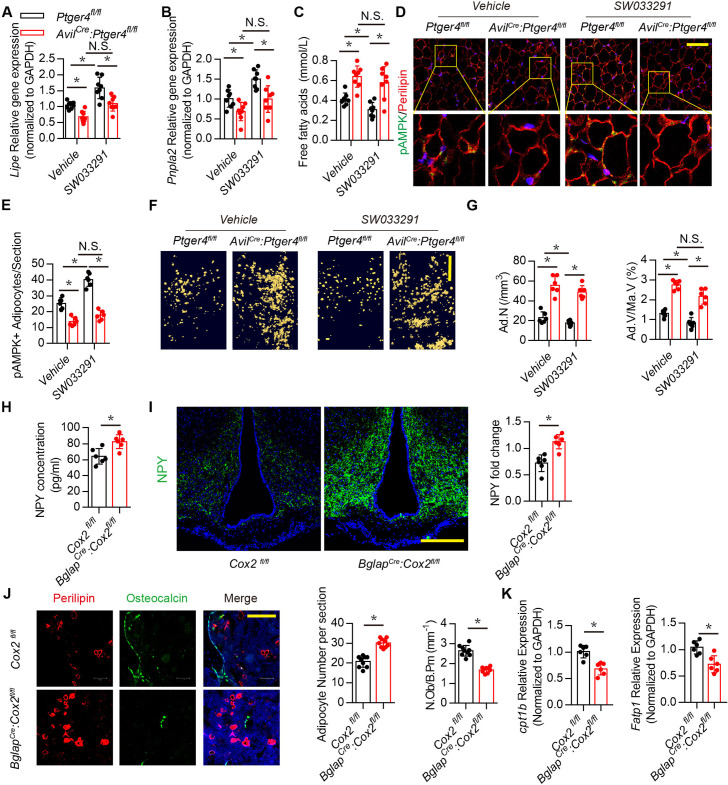


The original Figure 5:

**Figure 2:**
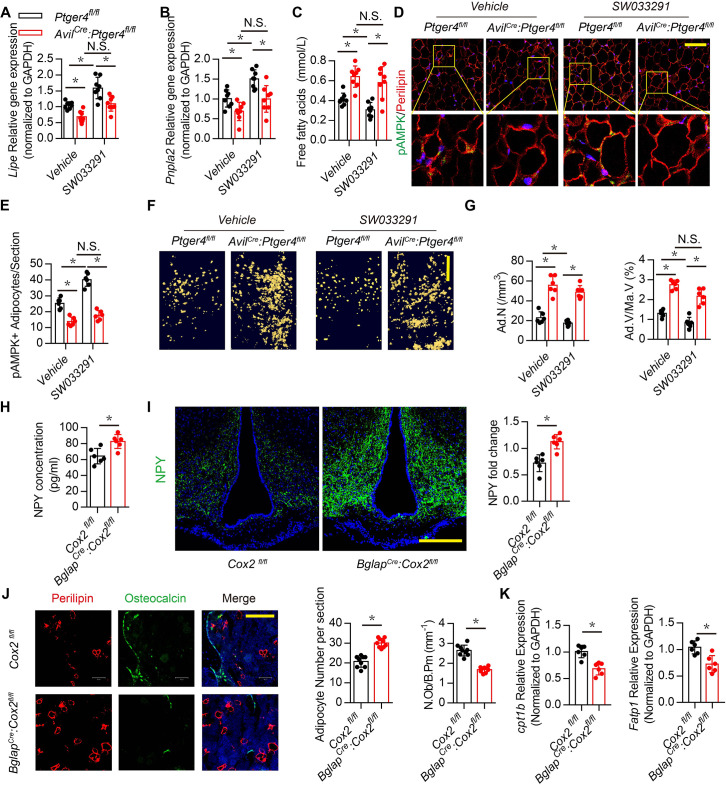


The corrected Figure 7 (Panel A):

**Figure 3:**
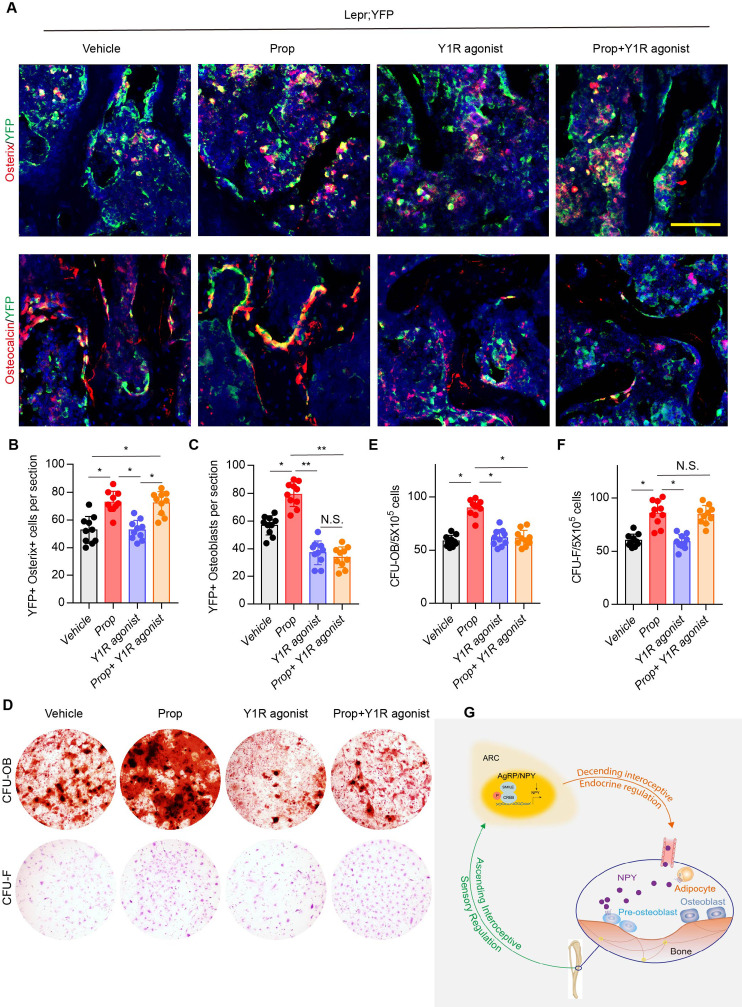


The original Figure7A:

**Figure 4:**
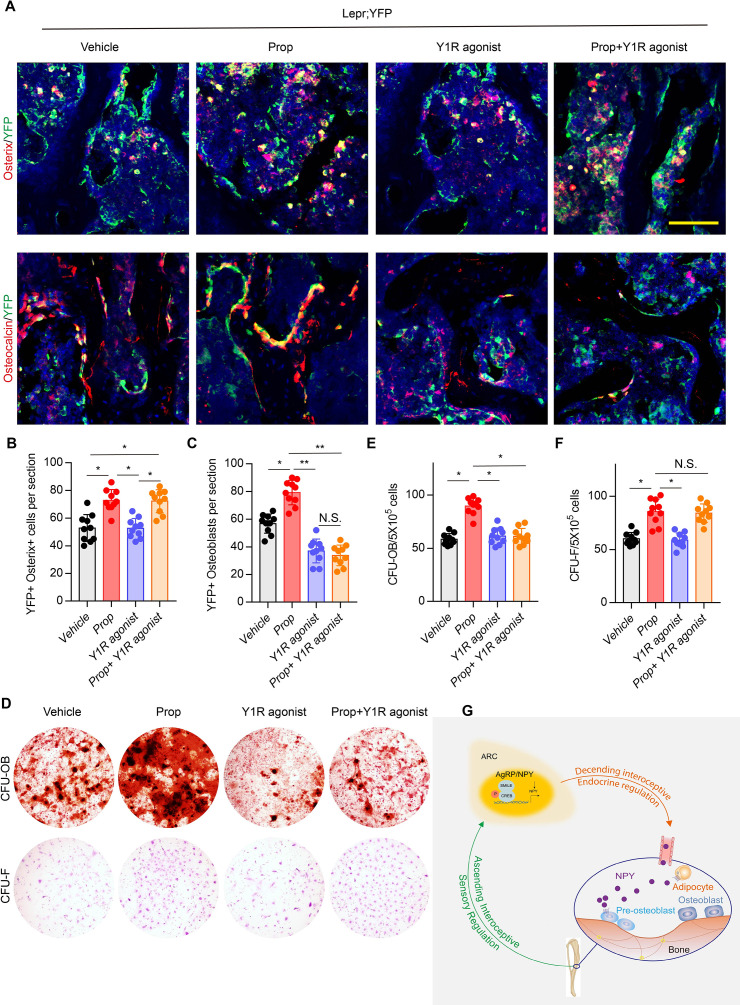


The article has been corrected accordingly.

