# Supplementary material for: Skeleton interoception regulates bone and fat metabolism through hypothalamic neuroendocrine NPY
Source: eLife. 2021 Sep 1;10:e70324. doi: 10.7554/eLife.70324 (PMC8439655; doi:10.7554/eLife.70324)
Supplement: Supplementary file 1. [file elife-70324-supp1.docx]

|  | Gene | Primer sequence (5’-3’) |
| --- | --- | --- |
| Transgenic mice genomic sequence | *Avil-Cre*: forward | CCCTGTTCACTGTGAGTAGG |
|  | *Avil-Cre*: reverse | GCGATCCCTGAACATGTCCATC |
|  | *Avil-Cre*: wt | AGTATCTGGTAGGTGCTTCCAG |
|  | *Ptger4* loxP allele forward | TCTGTGAAGCGAGTCCTTAGGCT |
|  | *Ptger4* loxP allele reverse | CGCACTCTCTCTCTCCCAAGGAA |
|  | *Ntrk1* loxP allele forward | AACAGTTTTGAGCATTTTCTATTGTTT |
|  | *Ntrk1* loxP allele reverse | CAAAGAAAACAGAAGAAAAATAATAC |
|  | *iDTR* loxP allele forward | GCGA AGAGTTTGTCCTCAACC |
|  | *iDTR* loxP allele reverse | AAAGTCGCTCTGAGTTGTTAT |
|  | *OCN-Cre*: forward | CAAATAGCCCTGGCAGATTC |
|  | *OCN-Cre*: reverse | TGATACAAGGGACATCTTCC |
|  | *DMP1-Cre* forward | TTGCCTTTCTCTCCACAGGT |
|  | *DMP1-Cre* reverse | CATGTCCATCAGGTTCTTGC |
|  | *Cox2* loxP allele forward | AATTACTGCTGAAGCCCACC |
|  | *Cox2* loxP allele forward | GAATCTCCTAGAACTGACTGG |
|  | *LepR-Cre* Mutant Forward | GCT GGA AGA TGG CGA TTA GC |
|  | *LepR-Cre* Wild type Forward | CCC AAT TTC AAA CCT GTT CC |
|  | *LepR-Cre* Common | TCT TCT TTC CAG AGT TCA GAT GT |
|  | *Rosa 26 EYFP* wt Forward | AAG GGA GCT GCA GTG GAG TA |
|  | *Rosa 26 EYFP* wt reverse | CCG AAA ATC TGT GGG AAG TC |
|  | *Rosa 26 EYFP* mutant forward | ACA TGG TCC TGC TGG AGT TC |
|  | *Rosa 26 EYFP* mutant reverse | GGC ATT AAA GCA GCG TAT CC |
| RT-PCR primers sequence | *Npy* forward | CAC GAT GCT AGG TAA CAA G |
|  | *Npy* reverse | CAC ATG GAA GGG TCT TCA AG |
|  | *Runx2* forward | TTACCTACACCCCGCCAGTC |
|  | *Runx2* reverse | TGCTGGTCTGGAAGGGTCC |
|  | *Alp* forward | ATCTTTGGTCTGGCTCCCATG |
|  | *Alp* reverse | TGAGCGACACGGACAAGAAGCCCTT |
|  | *Col1a1* forward | GACGCCATCAAGGTCTACTG |
|  | *Col1a1* reverse | ACGGGAATCCATCGGTCA |
|  | *Fatp1* forward | TGCTTTGGTTTCTGGGACTT |
|  | *Fapt1* reverse | GCTCTAGCCGAACACGAATC |
|  | *Cpt1b* forward | GGTCCCATAAGAAACAAGACCTC |
|  | *Cpt1b* reverse | AGACGATGTAAAGGGCAGAAGAGG |
|  | *Lipe* forward | CATCAACCACTGTGAGGGTAAG |
|  | *Lipe* reverse | AAGGGAGGTGAGATGGTAACT |
|  | *Pnpla2* forward | TAGCTAACAGTTGGGCTTCAC |
|  | *Pnpla2* reverse | CAGAGAGAACAGAGCAGCTTAC |
